# The Realization of a Remote Robotic Breast Surgery

**DOI:** 10.70352/scrj.cr.25-0607

**Published:** 2026-02-13

**Authors:** Fujun Shi, Jin Li, Wei Wei, Xiaohua Lin, Lizhen Zhu

**Affiliations:** 1 Department of Breast Surgery, Zhujiang Hospital of Southern Medical University, Guangzhou, Guangdong, China; 2 Ward 1, Department of General Surgery, Foshan Sanshui District People’s Hospital, Foshan, Guangdong, China

**Keywords:** remote surgery, breast cancer, robotic surgery, nipple-sparing mastectomy, 5G

## Abstract

**INTRODUCTION:**

While remote telesurgery has advanced fields like urology and general surgery, its application in breast oncology remains unreported. We present a successful case of remote robotic nipple-sparing mastectomy (rNSM), which preliminarily confirms its technical feasibility and perioperative safety.

**CASE PRESENTATION:**

A 50-year-old woman with multicentric, HER2-positive early-stage breast cancer accepted rNSM in January 2025. The surgical team operated from Zhujiang Hospital, controlling a single-port robotic system located 60 km away at Foshan Sanshui District People’s Hospital via a dedicated 5G standalone network. The procedure achieved R0 resection with minimal blood loss (<5 mL) and a mean network latency of 4.674 ms. Comprehensive contingency plans for network failure were in place. The patient recovered without complications.

**CONCLUSIONS:**

This case preliminarily establishes the technical feasibility and short-term safety of remote breast surgery in a controlled setting. It holds promise for improving access to specialized oncologic care. Future efforts must focus on establishing robust safety protocols, legal frameworks, and evaluating long-term oncological outcomes through larger studies.

## INTRODUCTION

Breast cancer is one of the most common malignancies among women worldwide. Surgical management has evolved from radical mastectomy toward minimally invasive techniques aimed at reducing morbidity and improving cosmesis. Laparoscopic and, subsequently, robotic-assisted techniques have been introduced to breast surgery, offering enhanced precision and improved recovery profile.^1)^ The recent advent of single-port (SP) robotic systems further minimizes invasiveness, allowing complex procedures like nipple-sparing mastectomy (NSM) to be performed through a single small incision.^2)^ Robotic NSM (rNSM), while associated with a learning curve, has been shown to potentially reduce complications such as nipple–areolar complex necrosis compared with conventional techniques.^3)^

Despite these advances, significant disparities persist in access to specialized surgical oncology care, particularly for patients in remote or underserved regions. Telesurgery—the ability to perform surgery at a distance—has emerged as a potential solution. Since the first transatlantic robotic cholecystectomy,^4)^ successful remote procedures have been reported in urology, neurosurgery, hepatobiliary-pancreatic surgery,^5–7)^ and so on. However, the application of telesurgery to breast oncology has not been described.

This case report details a successful remote rNSM for breast cancer. We describe the surgical setup, technological infrastructure, safety protocols, and short-term outcomes, and discuss the potential role and challenges of this approach within modern breast cancer care.

## CASE PRESENTATION

### Patient and preoperative assessment

**Fig. 1:**
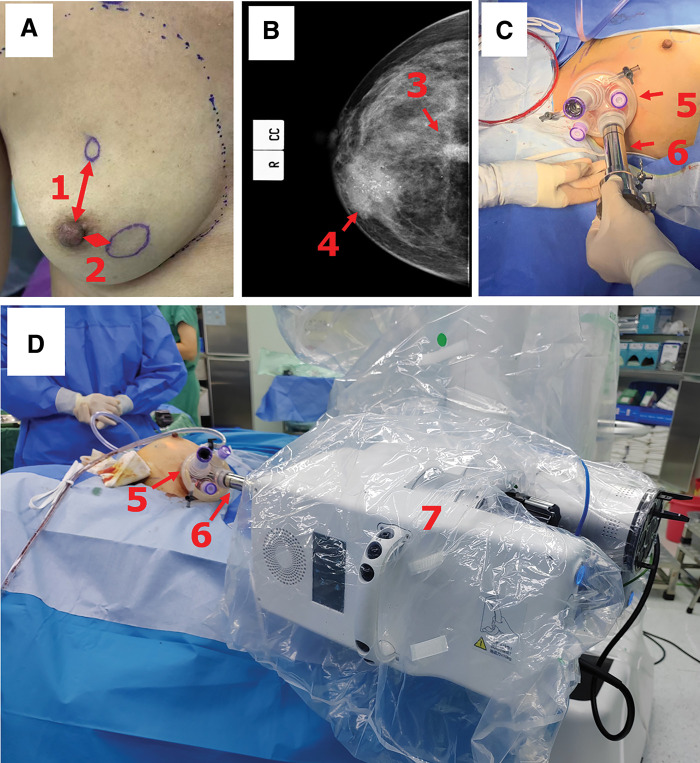
The scene at the patient’s side. (**A**) Preoperative photo of the patient’s right breast. There are two cancer nodules in the right breast confirmed by puncture pathology, located at the 12 o'clock and 4 o'clock directions, with distances from the nipple to be about 3.2 cm (1) and 1.2 cm (2), respectively. Neither of the cancerous areas has invaded the skin. (**B**) Preoperative mammography of the right breast. Number 3 refers to the mass at the 12 o'clock position, and number 4 refers to the mass at the 4 o'clock position, respectively. No evidence of intraductal extension was observed. (**C**) A right axillary incision was used to carry out sentinel lymph node biopsy, then the incision was separated subcutaneously. A single-port access device (5) was inserted and connected to a dedicated cannula of the robotic system (6). (**D**) The cannula was fixed onto the driving box (7), then medical carbon dioxide (10 mmHg) was insufflated into the setup cavity; the single-port patient cart was then set up for rNSM. rNSM, robot nipple-sparing mastectomy

The patient was a 50-year-old woman (BMI 24.9 kg/m^2^, breast size B Cup, mild breast ptosis [Regnault classification], no significant comorbidities), who presented with two palpable masses in her right breast (39 mm in largest diameter at 4 o’clock and 13 mm at 12 o’clock). Core needle biopsy confirmed multicentric invasive ductal carcinoma for both the sites while no axillary lymph node metastasis. Immunohistochemistry showed ER (−), PR (−), HER2 (3+), and Ki-67 60%. According to NCCN guidelines, she received neoadjuvant therapy (TCbHP regimen) for 6 cycles. Preoperative ultrasonography demonstrated that the tumors regressed to 19 mm at 4 o’clock and 11 mm at 12 o’clock, with a tumor-to-nipple distance of 1.2 and 3.2 cm, respectively; in addition, there was no evidence of skin or nipple–areolar complex involvement (**Fig. 1A**). The mammography (**Fig. 1B**) showed no evidence of intraductal extension, supporting the suitability for NSM.

The patient lived in a region with limited access to a high-volume breast cancer center. After detailed multidisciplinary discussion and thorough consultation regarding the novel remote procedure, she provided informed consent for a remote surgery, prioritizing the avoidance of extensive travel. After preoperational discussion with the patient, she opted for initial NSM surgery and a stage II implant reconstruction, so as to avoid complications such as capsular contracture after a possible postoperational radiotherapy.^8)^ The procedure was approved by the Institutional Ethics Committees of both participating hospitals (Approval No. 2023-JS-50-02).

### Surgical team and expertise

The primary surgeon was a board-certified breast surgeon with experience in over 30 rNSM procedures using the same robotic system and training in telesurgery protocols. This level of experience is consistent with reported learning curves for rNSM^9)^ and exceeds the suggested threshold for proficiency, aligning with expertise levels noted in reviews of robotic breast surgery.^3)^ The team at Sanshui has enough experience at open sentinel lymph node biopsy and open modified radical mastectomy.

### Remote surgical system and setup

**Fig. 2:**
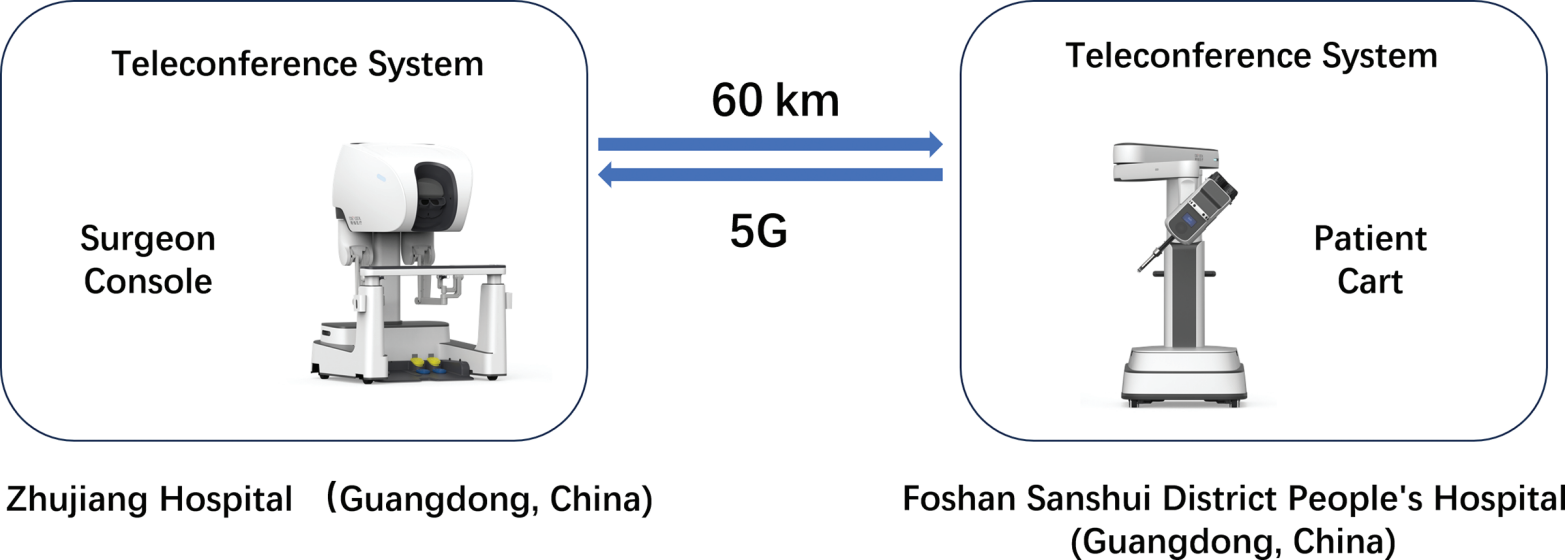
Illustration of the remote breast surgery. In this operation, a Surgeon console was set up at Zhujiang Hospital, a patient cart was set up at Foshan Sanshui District People’s Hospital, and the physical distance was 60 km. The 5G network architecture was applied to support this telesurgery, and teleconference systems enabled communications between the two groups.

The surgical setup is illustrated in **Fig. 2**.

**Surgeon Side (Zhujiang Hospital):** The primary surgeon and the surgeon’s console (Single-Port 1000, Edge Medical, Shenzhen, China; Software version 1.3.0.1).

**Patient Side (Foshan Sanshui District People’s Hospital, 60 km away):** The robotic patient cart (same as above), the patient, a senior assisting surgeon, an assistant, and nursing staff.

**Connectivity:** A hybrid network architecture was employed. The primary connection was a dedicated 5G standalone network with guaranteed bandwidth. A secondary, physically separate fiber-optic line served as a backup, with automatic switchover capability in case of 5G signal degradation or latency spike (>20 ms).

**Communication:** A secure, low-latency teleconferencing system enabled continuous audio-visual communication between the two sites.

### Contingency and safety protocol

A comprehensive safety protocol was established, aligning with recent guidelines for telesurgery^10)^: 1. Dedicated 5G standalone network failure: automatic switch to the backup fiber line. If both failed, the procedure would be paused immediately; 2. Local takeover: a fully credentialed breast surgeon, scrubbed and present at the patient’s side, was authorized to immediately take over and convert to standard open surgery if needed; 3. Instruments backup: standard open surgical instruments were available in the operating room.

### Operative technique

**Fig. 3:**
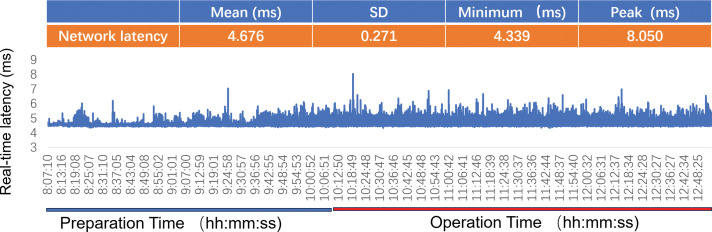
Network latency. Preparation time (blue bar) refers to the period from patient anesthesia and disinfection to the start of the surgery. During this time, we conduct tests on the remote surgical system by remotely operating a dedicated set of testing instruments, so as to verify their functionality and monitor network parameters (such as latency and bandwidth) in real time to ensure they meet the predefined surgical standards. Operation time (red bar) refers to the entire duration from the start to the end of the surgery. The table above summarizes the key statistical data of network latency during this process, including the average value, standard deviation, minimum latency, and peak latency.

First, the remote operability testing of the robotic system and network connection—including metrics such as network latency—was conducted, and the system was confirmed to be fully operational (**Fig. 3** Preparation Time). After general anesthesia, the patient was positioned supine with the right side elevated. The assisting team at Sanshui performed a 5-cm axillary incision and sentinel lymph node biopsy. As the pathological result of the nodes was negative on frozen section, axillary dissection was omitted. Through the same incision, subcutaneous dissection created a pocket, an SP access device (**Fig. 1B** [3]) was placed and connected to a dedicated cannula of the robotic system (**Fig. 1B** [4]), and the operative cavity was established with CO_2_ insufflation (10 mmHg). The patient cart was then docked (**Fig. 1C**).

**Fig. 4:**
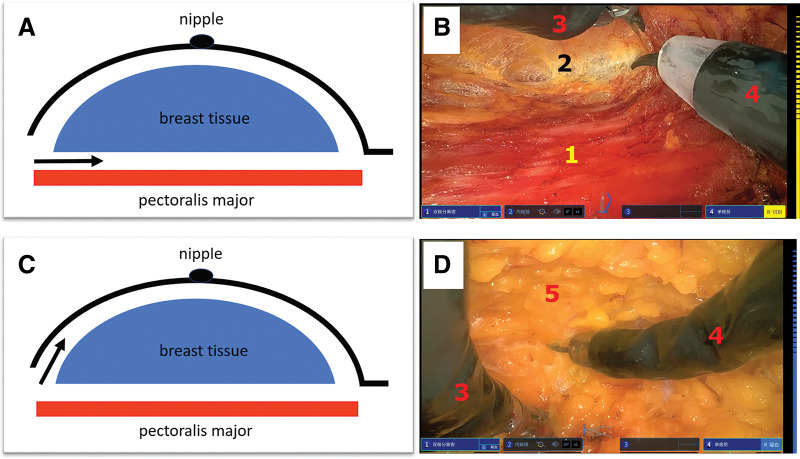
The process of the rNSM. The top two images illustrate the process of dissecting the retromammary space. (**A**) Schematic diagram, with the black arrow indicating the separation between the glandular tissue and the pectoralis major muscle. (**B**) An endoscopic view, where (1) indicates the pectoralis major muscle and (2) shows the pectoralis major fascia and glandular tissue. The bottom two images depict the process of dissecting the superficial layer of the glandular tissue. (**C**) Schematic diagram, with the black arrow indicating the separation between the glandular tissue and the subcutaneous tissue. (**D**) (5) represents the thin layer of skin with a small amount of adipose tissue. ([3] is the robotic system’s dissecting forceps and [4] is the robotic system’s monopolar scissors.) rNSM, robot nipple-sparing mastectomy

**Fig. 5:**
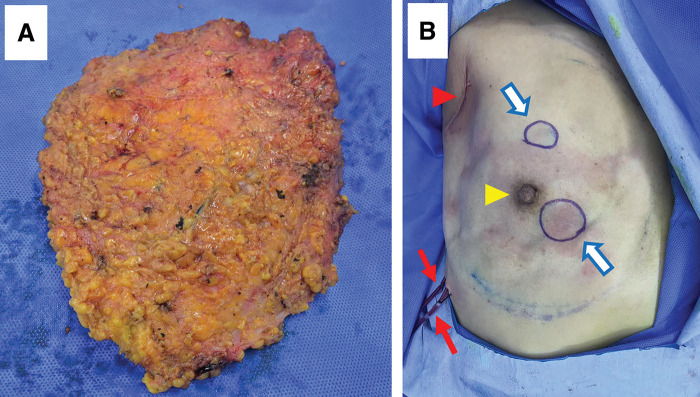
The remotely dissected right tissue and the breast post operation. (**A**) After the rNSM, the right breast tissue was taken out via the incision at the axilla, then two draining tubes (red arrows) were inserted and the axillary incision was sutured. (**B**) The two round blue circles (blue arrows) indicate the positions of the two breast tumors (Yellow arrowhead: the nipple; red arrowhead: the sutured axillary incision). rNSM, robot nipple-sparing mastectomy

The remote surgeon then performed the NSM using robotic bipolar dissector and monopolar scissors. The dissection proceeded systematically from below the pectoralis major fascia of the retromammary space (**Fig. 4A**, **4B**), to the superficial subcutaneous plane (**Figs. 4C**, **3D**), preserving a thin (no more than 5 mm), well-vascularized adipose layer over the skin flap. Meticulous remote hemostasis was achieved. The excised breast tissue was removed through the axillary incision (**Fig. 5A**). The intraoperative frozen section results of the subcutaneous tissue overlying the tumor, the pectoralis major tissue beneath the tumor base, and the retroareolar glandular tissue were all negative. Two drains were placed, and the incision was closed in layers by the on-site team (**Fig. 5B**); no intraoperative complications occurred during the whole surgical process. Because the intraoperative frozen section results were negative, two circles were marked on the original cancerous region, so as to ensure the corresponding areas could be accurately located for potential skin excision, in case the final postoperative pathology turned out to be positive (**Fig. 5B**).

### Intraoperative data and network performance

The total operative time was 162 minutes (sentinel lymph node biopsy: 29 min, docking: 8 min, console: 125 min). Intraoperative blood loss was less than 5 mL. Network latency was continuously monitored (**Fig. 3**). The latency during the console phase was 4.674 ms ± 0.271 ms, with a maximum recorded peak of 8.050 ms (Table in **Fig. 3**). No network disruptions or safety-critical events occurred. The surgeon reported no perceptible hindrance to instrument control. This latency is well below the thresholds reported to impact surgical performance^11)^ and is lower than most latencies reported in a recent systematic review of human telesurgery.^12)^

### Postoperative course and pathological findings

The patient’s recovery was uneventful. She was discharged on day 5 with minimal pain and excellent wound healing. Drains were removed on POD 7. At the 1-month follow-up, no complications were noted, and the patient reported high satisfaction with the cosmetic outcome and the travel-free surgical experience. The postoperative pathology report indicated that the neoadjuvant therapy achieved a complete pathological response, with no residual invasive carcinoma or ductal carcinoma in situ observed (Grade 5 in Miller-Payne Critia).

## DISCUSSION

This report describes a successful application of remote telesurgery to breast oncology. The integration of a high-stability 5G network, an robotic platform, and a rigorously planned safety protocol enabled the safe completion of an NSM across a 60-km distance.

### Technical feasibility and safety

NSM is applicable for breast management in breast cancer cases but carries specific eligibility requirements. Exclusion criteria included evidence of tumor involvement of the skin, evidence of tumor within 1 cm of the nipple–areolar complex, prior breast implants, breast size larger than D cup, and grade 3 nipple ptosis, among others.^13)^ To the case presented in this report, all the aforementioned criteria were fully met.

The cornerstone of successful telesurgery is ultralow latency and high reliability of the network. Our recorded latency (mean <5 ms) is consistent with the requirements for precise robotic surgery, as delays below 100–200 ms are generally considered non-critical for operator performance.^11)^ Notably, our latency is significantly lower than the range (28–280 ms) reported in a recent systematic review of human telesurgery across various specialties^12)^; also, throughout the entire procedure, the network latency exhibited minimal fluctuation, highlighting the satisfied state of the network infrastructure. The implementation of a redundant network and an on-site surgical safety net align with best-practice frameworks proposed for clinical telesurgery.^10)^ These measures effectively mitigate the primary risks associated with network dependency.

### Integration into breast cancer care pathways

The value of remote breast surgery extends beyond technical triumph. Its primary potential lies in democratizing access to high-quality, standardized surgical care within integrated treatment pathways. Patients in remote locations could receive neoadjuvant therapy locally, undergo remote surgery at a regional hub hospital performed by a centralized expert team, and continue adjuvant therapy and follow-up close to home. This hub and spoke model can help bridge the well-documented geographic disparities in cancer outcomes. Bibliometric analysis indicates that NSM and breast reconstruction are current research hotspots in robotic breast surgery,^14)^ and our remote surgery model directly facilitates the dissemination of these advanced techniques to underserved areas. However, the successful adoption of such models relies not only on technology but also on healthcare provider acceptance. Studies in resource-limited settings indicate that physicians’ knowledge and positive attitudes toward telesurgery are crucial for implementation and are influenced by factors like digital literacy and access to technology and training.^15)^ Our protocol development incorporated these considerations by ensuring team training and robust infrastructure.

### Ethical, legal, and logistical considerations

This nascent field necessitates parallel development of robust ethical and legal frameworks. Key issues include redefining concepts of the “operating theater” and “surgeon presence,” obtaining valid informed consent for a distributed-care model, and clarifying liability across jurisdictions. Our protocol, developed in consultation with hospital legal counsel, placed ultimate responsibility on the remote surgeon. This aligns with the “telesurgical support” model described in the Japanese clinical practice guidelines for telesurgery, where the local surgeon retains primary responsibility.^10)^ Future implementations should standardize liability agreements and consent processes.

### Limitations and future directions

This is a single-case report with short-term follow-up. It involved a highly selected patient and a seasoned surgical team in a controlled environment. The reported feasibility cannot be generalized without validation in larger, prospective studies. As Morrow (2021) critically notes, the oncological safety of robotic mastectomy remains unproven due to lack of long-term outcome data.^16)^ Our case adds to the initial experience but underscores the urgent need for such studies. Encouragingly, recent international multicenter data comparing rNSM to conventional NSM (CNSM) have shown rNSM to be associated with favorable surgical outcomes, including lower rates of nipple necrosis and major complications, without compromising short-to-medium-term oncologic safety^17)^ These findings, while not yet long-term, provide a stronger evidence base for the technical and perioperative safety of the robotic platform upon which our remote model is built. Future research on remote surgery must build upon this foundation. Future research must focus on: 1. Conducting comparative studies (remote vs. conventional rNSM) on operative metrics, complications, and cost; 2. Establishing universal technical standards for network reliability, latency thresholds, and fail-safe protocols; 3. Developing comprehensive legal and regulatory guidelines for cross-institutional telesurgery; and 4. Evaluating long-term oncological safety, patient-reported outcomes, and cost-effectiveness. Looking ahead, the convergence of telesurgery with advances in AI and surgical autonomy research^18)^ promises to further evolve these systems, potentially enhancing their capability, consistency, and impact.

## CONCLUSIONS

We have preliminarily demonstrated that remote rNSM is technically feasible and perioperatively safe when supported by appropriate technology, rigorous planning, and comprehensive safety protocols. While not intended to replace conventional surgery, remote breast surgery presents a transformative opportunity to improve equitable access to specialized oncologic care. Addressing the accompanying technological, legal, and logistical challenges through collaborative research is essential before broader clinical implementation can be considered.

## DECLARATIONS

### Availability of data and materials

Data are available from the corresponding author upon reasonable request.
